# Bis[(4-methyl­phen­yl)di­phenyl­phosphine-κ*P*](nitrito-κ^2^ *O*,*O*′)silver(I)

**DOI:** 10.1107/S2414314622007714

**Published:** 2022-08-12

**Authors:** Kariska Potgieter, Frederick P. Malan, Oyekunle Azeez Alimi, Reinout Meijboom

**Affiliations:** a Department of Chemical Sciences, University of Johannesburg, PO Box 524, Auckland Park, 2006, Johannesburg, South Africa; b Department of Chemistry, University of Pretoria, Lynnwood Road, Hatfield, Pretoria, 0002, South Africa; Sunway University, Malaysia

**Keywords:** silver(I) complex, diphenyl-*p*-tolyl­phosphine, nitrite, crystal structure

## Abstract

The title Ag^I^ complex, [Ag(NO_2_)(C_19_H_17_P)_2_], reveals a distorted pseudo-trigonal–planar shape around the Ag^I^ atom geometry resulting from the coordination of two phosphine ligands, as well as a nitrito-*O*,*O*′ ligand coordinating to the silver(I) atom through the oxygen atoms; in this description, the two oxygen atoms are assumed to occupy one position, forming an acute O—Ag—O angle of 51.44 (9)°. The plane resulting from the NO_2_ coordination to Ag is nearly perpendicular to the plane from the coordination of the phosphine-P atoms to Ag [dihedral angle = 86.43 (9)°].

## Abstract 2

The title silver(I) diphenyl-*p*-tolyl­phosphine complex crystallizes with one complete mol­ecule in the asymmetric unit that features a bidentate nitrito, as well as two diphenyl-*p*-tolyl­phosphine ligands coordinated to a Ag^I^ center.

## Graphical abstract

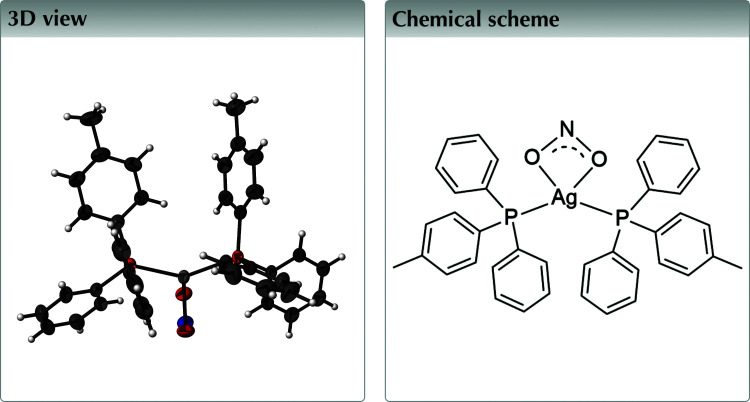

## Structure description

**Figure 1:**
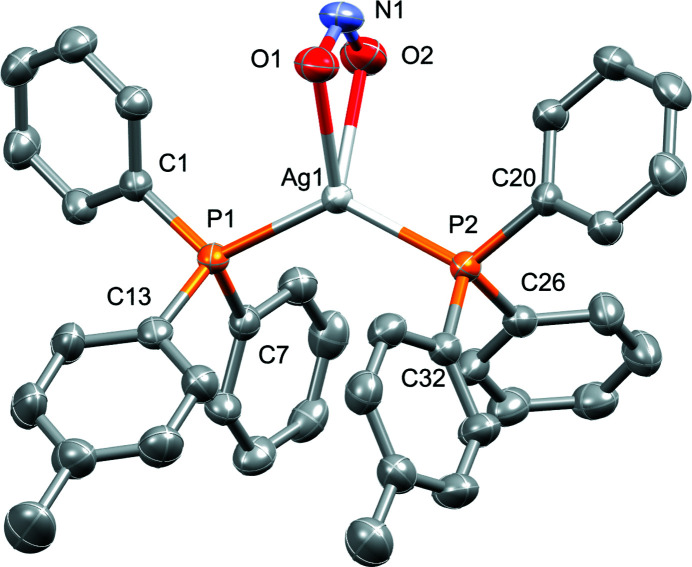
Perspective view of the mol­ecular structure of the title compound showing displacement ellipsoids at the 50% probability level. Hydrogen atoms are omitted for clarity.

**Figure 2:**
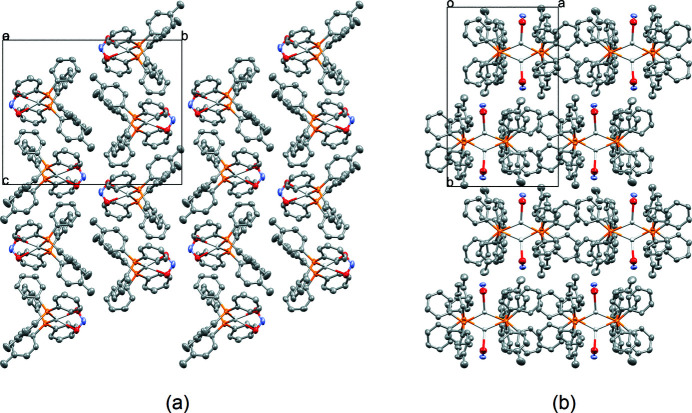
Packing diagrams as viewed along the (*a*) *a* and (*b*) *c* axes. Hydrogen atoms are omitted for clarity.

The mol­ecular structure of the title compound is shown in Fig. 1 ▸. The complex crystallizes in the monoclinic space group *P*2_1_/*c* with *Z* = 4. The asymmetric unit contains one complete silver complex mol­ecule, featuring an Ag^I^ atom, two diphenyl-*p*-tolyl­phosphine ligands, and one NO_2_ coordinating in a bidentate fashion. Near-identical Ag—P bond lengths are observed [Ag1—P1 = 2.4209 (7) Å and Ag1—P2 = 2.4251 (8) Å]. The nitrito ligand is similarly coordinating in a near symmetric fashion (Ag1—O1 = 2.422 (2), Ag1—O2 = 2.415 (2), N1—O1 = 1.253 (4) and N1–O2 = 1.255 (4) Å). As seen in Fig. 1 ▸, the four-coordinate silver(I) atom essentially exhibits a pseudo trigonal–planar shape with the three coordinating ligands, with bond angles P1—Ag1—P2 [129.51 (3)°], P1—Ag1—O1 [116.23 (7)°], P1—Ag1—O2 [111.09 (7)°], P2—Ag1—O1 [110.79 (7)°], P2—Ag1—O2 [111.96 (7)°], and O1—Ag1—O2 [51.44 (9)°]; in this description, the two oxygen atoms are assumed to occupy one position. The plane Pl1 defined by Ag1, O1, O2 and N1 crosses the plane Pl2 defined by P1, P2 and Ag1 at an angle of 86.43 (9)°. The *ipso*-carbon atoms of each of the phosphine ligands overlap in a near-eclipsed fashion when viewed down the P1—Ag1—P2 plane Pl2. Corresponding torsion angles are Ag1—P1—C1—C2 = −23.4 (3)°, Ag1—P1—C7—C8 = −51.9 (3)°, Ag1—P1—C13—C14 = 147.8 (3)°, Ag1—P2—C20—C21 = −29.0 (3)°, Ag1—P2—C26—C27 = 133.3 (3) and Ag1—P2—C32—C33 = 132.3 (3)°. The complex packs in three dimensions as layers of mol­ecules, leaving thin corrugated channels in between the inorganic layers when viewed along the *a* axis (Fig. 2 ▸).

## Synthesis and crystallization

Diphenyl-*p*-tolyl­phosphine (1 mmol) was dissolved in aceto­nitrile (10 ml). Silver nitrite (1 mmol) was dissolved in aceto­nitrile (5 ml). The diphenyl-*p*-tolyl­phosphine solution (10 ml) was added to the silver nitrite solution (5 ml), to give a 2:1 molar ratio reaction. The mixture was heated under reflux for 2 h after which the solution was left to crystallize.

## Refinement

For full experimental details including crystal data, data collection and structure refinement details, refer to Table 1 ▸.

## Supplementary Material

Crystal structure: contains datablock(s) I. DOI: 10.1107/S2414314622007714/tk4082sup1.cif

Structure factors: contains datablock(s) I. DOI: 10.1107/S2414314622007714/tk4082Isup2.hkl

Supporting information file. DOI: 10.1107/S2414314622007714/tk4082Isup3.cdx

CCDC reference: 2193913

Additional supporting information:  crystallographic information; 3D view; checkCIF report

## Figures and Tables

**Table 1:** Experimental details

Crystal data	
Chemical formula	[Ag(NO_2_)(C_19_H_17_P)_2_]
*M*_r_	706.47
Crystal system, space group	Monoclinic, *P*2_1_/*c*
Temperature (K)	150
*a*, *b*, *c* (Å)	11.8709 (2), 18.6292 (2), 15.4003 (2)
β (°)	103.055 (1)
*V* (Å^3^)	3317.68 (8)
*Z*	4
Radiation type	Cu *K*α
μ (mm^−1^)	6.05
Crystal size (mm)	0.24 × 0.13 × 0.10

Data collection	
Diffractometer	XtaLAB Synergy R, DW system, HyPix
Absorption correction	Multi-scan (*CrysAlis PRO*; Rigaku OD, 2022 ▸)
*T*_min_, *T*_max_	0.188, 1.000
No. of measured, independent and observed [*I* > 2σ(*I*)] reflections	41716, 7030, 6535
*R*_int_	0.049
(sin θ/λ)_max_ (Å^−1^)	0.637

Refinement	
*R*[*F* ^2^ > 2σ(*F* ^2^)], *wR*(*F* ^2^), *S*	0.040, 0.104, 1.07
No. of reflections	7030
No. of parameters	399
H-atom treatment	H-atom parameters constrained
Δρ_max_, Δρ_min_ (e Å^−3^)	0.68, −0.82

Computer programs: *CrysAlis PRO* (Rigaku OD, 2022 ▸), *SHELXT* (Sheldrick, 2015*b* ▸), *SHELXL* (Sheldrick, 2015*a* ▸), and *OLEX2* (Dolomanov *et al.*, 2009 ▸).

## full crystallographic data

### Crystal data

**Table:**

[Ag(NO_2_)(C_19_H_17_P)_2_]	*F*(000) = 1448
*M**_r_* = 706.47	*D*_x_ = 1.414 Mg m^−^^3^
Monoclinic, *P*2_1_/*c*	Cu *K*α radiation, λ = 1.54184 Å
*a* = 11.8709 (2) Å	Cell parameters from 29792 reflections
*b* = 18.6292 (2) Å	θ = 3.8–78.9°
*c* = 15.4003 (2) Å	µ = 6.05 mm^−^^1^
β = 103.055 (1)°	*T* = 150 K
*V* = 3317.68 (8) Å^3^	Block, colourless
*Z* = 4	0.24 × 0.13 × 0.10 mm

### Data collection

**Table:**

XtaLAB Synergy R, DW system, HyPix diffractometer	7030 independent reflections
Radiation source: Rotating-anode X-ray tube, Rigaku (Cu) X-ray Source	6535 reflections with *I* > 2σ(*I*)
Mirror monochromator	*R*_int_ = 0.049
Detector resolution: 10.0000 pixels mm^-1^	θ_max_ = 79.2°, θ_min_ = 3.8°
ω scans	*h* = −14→15
Absorption correction: multi-scan (CrysAlisPro; Rigaku OD, 2022)	*k* = −23→23
*T*_min_ = 0.188, *T*_max_ = 1.000	*l* = −18→19
41716 measured reflections	

### Refinement

**Table:**

Refinement on *F*^2^	Primary atom site location: dual
Least-squares matrix: full	Hydrogen site location: inferred from neighbouring sites
*R*[*F*^2^ > 2σ(*F*^2^)] = 0.040	H-atom parameters constrained
*wR*(*F*^2^) = 0.104	*w* = 1/[σ^2^(*F*_o_^2^) + (0.0425*P*)^2^ + 5.6399*P*] where *P* = (*F*_o_^2^ + 2*F*_c_^2^)/3
*S* = 1.07	(Δ/σ)_max_ = 0.002
7030 reflections	Δρ_max_ = 0.68 e Å^−^^3^
399 parameters	Δρ_min_ = −0.82 e Å^−^^3^
0 restraints	

### Special details

**Table:**

Geometry. All esds (except the esd in the dihedral angle between two l.s. planes) are estimated using the full covariance matrix. The cell esds are taken into account individually in the estimation of esds in distances, angles and torsion angles; correlations between esds in cell parameters are only used when they are defined by crystal symmetry. An approximate (isotropic) treatment of cell esds is used for estimating esds involving l.s. planes.
Refinement. All H atoms were placed in geometrically idealized positions and constrained to ride on their parent atoms.

### Fractional atomic coordinates and isotropic or equivalent isotropic displacement parameters (Å^2^)

**Table:**

	*x*	*y*	*z*	*U*_iso_*/*U*_eq_	
Ag1	0.33615 (2)	0.79867 (2)	0.56752 (2)	0.03178 (8)	
P1	0.52794 (6)	0.74875 (4)	0.61894 (5)	0.02991 (16)	
P2	0.14991 (6)	0.73885 (4)	0.53633 (6)	0.03298 (17)	
O1	0.3244 (2)	0.91204 (13)	0.48903 (17)	0.0459 (6)	
O2	0.3250 (2)	0.91839 (14)	0.62523 (18)	0.0506 (6)	
N1	0.3202 (3)	0.95191 (15)	0.5538 (2)	0.0492 (8)	
C7	0.5331 (3)	0.69367 (17)	0.7177 (2)	0.0325 (6)	
C1	0.6466 (3)	0.81224 (16)	0.6530 (2)	0.0321 (6)	
C25	−0.0805 (3)	0.77034 (18)	0.4467 (2)	0.0380 (7)	
H25	−0.0857	0.7224	0.4253	0.046*	
C20	0.0223 (3)	0.79524 (16)	0.5000 (2)	0.0334 (6)	
C26	0.1234 (3)	0.69196 (16)	0.6342 (2)	0.0355 (7)	
C13	0.5720 (3)	0.68644 (16)	0.5419 (2)	0.0337 (6)	
C6	0.7500 (3)	0.79432 (18)	0.7117 (2)	0.0385 (7)	
H6	0.7610	0.7473	0.7360	0.046*	
C24	−0.1757 (3)	0.8154 (2)	0.4248 (2)	0.0443 (8)	
H24	−0.2462	0.7980	0.3888	0.053*	
C32	0.1398 (3)	0.66934 (17)	0.4515 (2)	0.0360 (7)	
C2	0.6317 (3)	0.88145 (17)	0.6186 (2)	0.0352 (7)	
H2	0.5610	0.8943	0.5790	0.042*	
C8	0.4942 (3)	0.7238 (2)	0.7887 (2)	0.0413 (7)	
H8	0.4726	0.7729	0.7870	0.050*	
C37	0.1822 (3)	0.68542 (19)	0.3764 (2)	0.0425 (8)	
H37	0.2095	0.7325	0.3690	0.051*	
C31	0.2138 (3)	0.65361 (19)	0.6870 (3)	0.0456 (8)	
H31	0.2859	0.6510	0.6702	0.055*	
C5	0.8371 (3)	0.8450 (2)	0.7349 (3)	0.0461 (8)	
H5	0.9076	0.8326	0.7750	0.055*	
C18	0.4881 (3)	0.64259 (19)	0.4912 (3)	0.0440 (8)	
H18	0.4096	0.6485	0.4941	0.053*	
C10	0.5184 (3)	0.6103 (2)	0.8646 (2)	0.0459 (8)	
H10	0.5119	0.5816	0.9143	0.055*	
C12	0.5662 (3)	0.62220 (17)	0.7224 (2)	0.0374 (7)	
H12	0.5942	0.6014	0.6750	0.045*	
C14	0.6851 (3)	0.6784 (2)	0.5339 (3)	0.0474 (8)	
H14	0.7437	0.7089	0.5665	0.057*	
C11	0.5589 (3)	0.5806 (2)	0.7959 (2)	0.0447 (8)	
H11	0.5819	0.5317	0.7985	0.054*	
C4	0.8218 (3)	0.9132 (2)	0.7003 (2)	0.0453 (8)	
H4	0.8817	0.9478	0.7167	0.054*	
C3	0.7192 (3)	0.93168 (19)	0.6415 (2)	0.0445 (8)	
H3	0.7090	0.9787	0.6171	0.053*	
C23	−0.1685 (3)	0.8854 (2)	0.4552 (3)	0.0457 (8)	
H23	−0.2335	0.9164	0.4398	0.055*	
C21	0.0295 (3)	0.86579 (18)	0.5299 (3)	0.0447 (8)	
H21	0.1000	0.8837	0.5653	0.054*	
C27	0.0206 (3)	0.6970 (2)	0.6615 (3)	0.0491 (9)	
H27	−0.0422	0.7235	0.6271	0.059*	
C29	0.0976 (4)	0.6233 (2)	0.7899 (3)	0.0515 (9)	
H29	0.0878	0.5992	0.8421	0.062*	
C35	0.1463 (3)	0.5644 (2)	0.3217 (3)	0.0482 (8)	
C9	0.4873 (3)	0.6817 (2)	0.8616 (2)	0.0492 (9)	
H9	0.4609	0.7023	0.9098	0.059*	
C22	−0.0663 (3)	0.9098 (2)	0.5080 (3)	0.0518 (9)	
H22	−0.0615	0.9577	0.5297	0.062*	
C36	0.1850 (3)	0.6340 (2)	0.3126 (3)	0.0465 (8)	
H36	0.2137	0.6462	0.2617	0.056*	
C30	0.2004 (3)	0.6192 (2)	0.7633 (3)	0.0520 (9)	
H30	0.2628	0.5924	0.7978	0.062*	
C17	0.5172 (4)	0.5902 (2)	0.4363 (3)	0.0523 (9)	
H17	0.4585	0.5601	0.4028	0.063*	
C33	0.0991 (3)	0.60034 (19)	0.4599 (3)	0.0488 (9)	
H33	0.0689	0.5882	0.5101	0.059*	
C15	0.7132 (4)	0.6261 (2)	0.4787 (3)	0.0580 (11)	
H15	0.7914	0.6210	0.4744	0.070*	
C16	0.6305 (4)	0.5809 (2)	0.4296 (3)	0.0542 (10)	
C28	0.0095 (4)	0.6631 (2)	0.7393 (3)	0.0579 (10)	
H28	−0.0611	0.6676	0.7581	0.069*	
C34	0.1023 (4)	0.5492 (2)	0.3954 (3)	0.0568 (10)	
H34	0.0735	0.5025	0.4019	0.068*	
C38	0.1539 (4)	0.5072 (3)	0.2557 (3)	0.0684 (12)	
H38A	0.0815	0.5056	0.2100	0.103*	
H38B	0.1668	0.4607	0.2860	0.103*	
H38C	0.2183	0.5177	0.2275	0.103*	
C19	0.6634 (5)	0.5233 (3)	0.3706 (4)	0.0806 (16)	
H19A	0.6400	0.4763	0.3889	0.121*	
H19B	0.7473	0.5239	0.3763	0.121*	
H19C	0.6242	0.5325	0.3085	0.121*	

### Atomic displacement parameters (Å^2^)

**Table:**

	*U*^11^	*U*^22^	*U*^33^	*U*^12^	*U*^13^	*U*^23^
Ag1	0.02537 (12)	0.02500 (12)	0.04510 (14)	−0.00004 (7)	0.00826 (9)	0.00223 (8)
P1	0.0263 (3)	0.0251 (3)	0.0388 (4)	0.0012 (3)	0.0084 (3)	0.0051 (3)
P2	0.0250 (3)	0.0251 (4)	0.0489 (5)	−0.0007 (3)	0.0084 (3)	0.0014 (3)
O1	0.0571 (15)	0.0328 (12)	0.0506 (14)	0.0036 (11)	0.0179 (12)	0.0019 (10)
O2	0.0616 (16)	0.0403 (14)	0.0527 (15)	−0.0017 (12)	0.0191 (12)	−0.0120 (12)
N1	0.0524 (18)	0.0248 (14)	0.071 (2)	0.0032 (12)	0.0154 (15)	−0.0042 (14)
C7	0.0261 (14)	0.0343 (16)	0.0376 (16)	−0.0021 (11)	0.0080 (12)	0.0028 (12)
C1	0.0288 (14)	0.0299 (15)	0.0390 (16)	0.0013 (12)	0.0104 (12)	0.0028 (12)
C25	0.0315 (16)	0.0320 (16)	0.0496 (19)	0.0001 (12)	0.0075 (13)	−0.0020 (14)
C20	0.0252 (14)	0.0295 (15)	0.0462 (17)	−0.0008 (11)	0.0098 (12)	0.0019 (12)
C26	0.0319 (15)	0.0267 (14)	0.0482 (18)	−0.0009 (12)	0.0097 (13)	−0.0024 (13)
C13	0.0364 (16)	0.0284 (14)	0.0373 (16)	0.0020 (12)	0.0105 (13)	0.0086 (12)
C6	0.0302 (16)	0.0354 (17)	0.0488 (19)	0.0031 (12)	0.0064 (13)	0.0082 (14)
C24	0.0332 (17)	0.051 (2)	0.0455 (19)	0.0061 (15)	0.0027 (14)	0.0003 (16)
C32	0.0291 (15)	0.0294 (15)	0.0499 (18)	0.0013 (12)	0.0094 (13)	0.0004 (13)
C2	0.0336 (16)	0.0323 (15)	0.0381 (16)	0.0010 (12)	0.0049 (12)	0.0034 (12)
C8	0.0414 (18)	0.0389 (17)	0.0440 (18)	−0.0020 (14)	0.0107 (14)	−0.0029 (14)
C37	0.0407 (18)	0.0345 (17)	0.052 (2)	−0.0064 (14)	0.0102 (15)	0.0017 (15)
C31	0.0332 (17)	0.0369 (17)	0.068 (2)	−0.0016 (14)	0.0136 (16)	0.0122 (16)
C5	0.0319 (16)	0.053 (2)	0.051 (2)	−0.0049 (15)	0.0037 (14)	0.0041 (16)
C18	0.0378 (18)	0.0388 (18)	0.056 (2)	0.0015 (14)	0.0121 (15)	−0.0036 (15)
C10	0.0444 (19)	0.054 (2)	0.0378 (18)	−0.0127 (16)	0.0067 (14)	0.0094 (15)
C12	0.0369 (16)	0.0339 (16)	0.0428 (17)	0.0064 (13)	0.0119 (13)	0.0091 (13)
C14	0.0400 (19)	0.046 (2)	0.063 (2)	−0.0086 (15)	0.0252 (17)	−0.0084 (17)
C11	0.0467 (19)	0.0419 (19)	0.0436 (19)	−0.0007 (15)	0.0064 (15)	0.0128 (15)
C4	0.0398 (18)	0.0428 (19)	0.053 (2)	−0.0134 (15)	0.0101 (15)	−0.0008 (16)
C3	0.0465 (19)	0.0351 (17)	0.050 (2)	−0.0054 (14)	0.0082 (15)	0.0064 (15)
C23	0.0373 (18)	0.0406 (18)	0.059 (2)	0.0137 (14)	0.0114 (15)	0.0070 (16)
C21	0.0310 (16)	0.0308 (16)	0.071 (2)	−0.0017 (13)	0.0083 (15)	−0.0048 (15)
C27	0.0391 (19)	0.050 (2)	0.062 (2)	0.0066 (16)	0.0194 (17)	0.0084 (17)
C29	0.068 (3)	0.0368 (18)	0.054 (2)	−0.0029 (17)	0.0217 (19)	0.0043 (16)
C35	0.0433 (19)	0.0421 (19)	0.061 (2)	0.0021 (15)	0.0152 (17)	−0.0068 (17)
C9	0.049 (2)	0.064 (2)	0.0366 (18)	−0.0079 (18)	0.0137 (15)	−0.0050 (16)
C22	0.044 (2)	0.0314 (17)	0.081 (3)	0.0053 (15)	0.0172 (19)	−0.0036 (17)
C36	0.0430 (19)	0.052 (2)	0.048 (2)	−0.0034 (16)	0.0178 (16)	0.0013 (16)
C30	0.047 (2)	0.0399 (19)	0.067 (2)	−0.0023 (16)	0.0076 (18)	0.0153 (17)
C17	0.056 (2)	0.046 (2)	0.057 (2)	−0.0090 (17)	0.0154 (18)	−0.0139 (17)
C33	0.058 (2)	0.0320 (17)	0.062 (2)	−0.0083 (16)	0.0264 (18)	−0.0040 (16)
C15	0.052 (2)	0.056 (2)	0.078 (3)	−0.0100 (19)	0.040 (2)	−0.016 (2)
C16	0.069 (3)	0.044 (2)	0.059 (2)	−0.0045 (19)	0.033 (2)	−0.0089 (17)
C28	0.057 (2)	0.056 (2)	0.071 (3)	0.0122 (19)	0.036 (2)	0.012 (2)
C34	0.069 (3)	0.0321 (18)	0.076 (3)	−0.0061 (17)	0.032 (2)	−0.0040 (18)
C38	0.071 (3)	0.065 (3)	0.076 (3)	0.001 (2)	0.030 (2)	−0.014 (2)
C19	0.094 (4)	0.072 (3)	0.090 (4)	−0.013 (3)	0.051 (3)	−0.037 (3)

### Geometric parameters (Å, º)

**Table:**

Ag1—P1	2.4209 (7)	C32—C37	1.395 (5)
Ag1—P2	2.4251 (8)	C32—C33	1.390 (5)
Ag1—O1	2.422 (2)	C2—C3	1.383 (5)
Ag1—O2	2.415 (2)	C8—C9	1.386 (5)
P1—C7	1.825 (3)	C37—C36	1.377 (5)
P1—C1	1.823 (3)	C31—C30	1.380 (5)
P1—C13	1.820 (3)	C5—C4	1.373 (5)
P2—C20	1.824 (3)	C18—C17	1.385 (5)
P2—C26	1.830 (3)	C10—C11	1.373 (5)
P2—C32	1.824 (3)	C10—C9	1.380 (6)
O1—N1	1.253 (4)	C12—C11	1.389 (5)
O2—N1	1.255 (4)	C14—C15	1.382 (5)
C7—C8	1.396 (5)	C4—C3	1.387 (5)
C7—C12	1.386 (4)	C23—C22	1.377 (5)
C1—C6	1.391 (4)	C21—C22	1.380 (5)
C1—C2	1.390 (4)	C27—C28	1.386 (6)
C25—C20	1.388 (4)	C29—C30	1.374 (6)
C25—C24	1.387 (5)	C29—C28	1.374 (6)
C20—C21	1.389 (4)	C35—C36	1.394 (5)
C26—C31	1.388 (5)	C35—C34	1.381 (6)
C26—C27	1.381 (5)	C35—C38	1.489 (6)
C13—C18	1.384 (5)	C17—C16	1.383 (6)
C13—C14	1.384 (5)	C33—C34	1.383 (5)
C6—C5	1.386 (5)	C15—C16	1.381 (6)
C24—C23	1.381 (5)	C16—C19	1.513 (6)
			
P1—Ag1—P2	129.51 (3)	C5—C6—C1	120.1 (3)
P1—Ag1—O1	116.23 (7)	C23—C24—C25	120.3 (3)
O1—Ag1—P2	110.79 (7)	C37—C32—P2	117.7 (2)
O2—Ag1—P1	111.09 (7)	C33—C32—P2	123.9 (3)
O2—Ag1—P2	111.96 (7)	C33—C32—C37	118.3 (3)
O2—Ag1—O1	51.44 (9)	C3—C2—C1	120.4 (3)
C7—P1—Ag1	109.92 (10)	C9—C8—C7	119.8 (3)
C1—P1—Ag1	116.95 (10)	C36—C37—C32	120.9 (3)
C1—P1—C7	104.28 (14)	C30—C31—C26	121.0 (3)
C13—P1—Ag1	114.79 (11)	C4—C5—C6	120.3 (3)
C13—P1—C7	103.00 (14)	C13—C18—C17	120.9 (3)
C13—P1—C1	106.52 (14)	C11—C10—C9	119.9 (3)
C20—P2—Ag1	116.91 (10)	C7—C12—C11	120.6 (3)
C20—P2—C26	104.02 (15)	C15—C14—C13	120.3 (4)
C26—P2—Ag1	112.02 (11)	C10—C11—C12	120.1 (3)
C32—P2—Ag1	112.17 (10)	C5—C4—C3	120.2 (3)
C32—P2—C20	105.88 (15)	C2—C3—C4	119.8 (3)
C32—P2—C26	104.80 (15)	C22—C23—C24	119.4 (3)
N1—O1—Ag1	97.3 (2)	C22—C21—C20	119.8 (3)
N1—O2—Ag1	97.59 (19)	C26—C27—C28	119.7 (4)
O1—N1—O2	113.6 (3)	C28—C29—C30	118.3 (4)
C8—C7—P1	118.2 (2)	C36—C35—C38	121.7 (4)
C12—C7—P1	122.7 (3)	C34—C35—C36	117.8 (3)
C12—C7—C8	119.0 (3)	C34—C35—C38	120.5 (4)
C6—C1—P1	122.8 (2)	C10—C9—C8	120.6 (3)
C2—C1—P1	117.9 (2)	C23—C22—C21	121.0 (3)
C2—C1—C6	119.2 (3)	C37—C36—C35	121.0 (3)
C24—C25—C20	120.1 (3)	C29—C30—C31	120.6 (4)
C25—C20—P2	123.2 (2)	C16—C17—C18	121.0 (4)
C25—C20—C21	119.4 (3)	C34—C33—C32	120.2 (4)
C21—C20—P2	117.3 (2)	C16—C15—C14	121.7 (4)
C31—C26—P2	118.3 (3)	C17—C16—C19	121.4 (4)
C27—C26—P2	123.1 (3)	C15—C16—C17	117.8 (4)
C27—C26—C31	118.4 (3)	C15—C16—C19	120.8 (4)
C18—C13—P1	117.9 (3)	C29—C28—C27	121.8 (4)
C14—C13—P1	123.7 (3)	C35—C34—C33	121.8 (4)
C14—C13—C18	118.3 (3)		
